# Effects of Temperature and Humidity on Soil Gross Nitrogen Transformation in a Typical Shrub Ecosystem in Yanshan Mountain and Hilly Region

**DOI:** 10.3390/life13030643

**Published:** 2023-02-25

**Authors:** Xiaoxia Hu, Yuanxun Zhang, Dong Wang, Jian Ma, Kaibing Xue, Zhaobo An, Wenxing Luo, Yizhi Sheng

**Affiliations:** 1 College of Resources and Environment, University of Chinese Academy of Sciences, Beijing 100049, China; 2 Beijing Yanshan Earth Critical Zone National Research Station, University of Chinese Academy of Sciences, Beijing 101408, China; 3 Institute of Eco-Environmental Forensics, Shandong University, Qingdao 266237, China; 4 National Marine Data and Information Service, Tianjin 300171, China; 5 College of Life Sciences, University of Chinese Academy of Sciences, Beijing 100049, China; 6 Center for Geomicrobiology and Biogeochemistry Research, State Key Laboratory of Biogeology and Environmental Geology, China University of Geosciences, Beijing 100083, China

**Keywords:** gross ammonification, gross nitrification, shrubland, soil temperature, soil moisture content

## Abstract

Shrubland is a pivotal terrestrial ecosystem in China. Soil nitrogen transformations play a crucial role in maintaining the productivity of this ecosystem, yet the driving forces underlying it have not been sufficiently addressed, particularly under ongoing climate changes. Herein, by incorporating ^15^N isotope pool dilution method in laboratory incubation, the rates of gross N ammonification, nitrification, and inorganic N consumption in soils in response to varying temperature and humidity conditions were determined at different depths (SL10: 0–10 cm, and SL20: 10–20 cm) in a typical shrub ecosystem in the Yanshan mountain and hilly region, North China. The gross rates of ammonification and nitrification of soils in SL10 were higher than those in SL20, which was likely affected by the higher soil organic matter and total N contents at a shallower depth. Both temperature and humidity significantly affected the N transformations. The gross ammonification and nitrification were significantly stimulated as the incubation temperature increased from 5 to 35 °C. The gross ammonification increased exponentially, while the gross nitrification increased differently in different temperature ranges. The increment of soil water contents (from 30% WHC to 60% and 100% WHC) promoted the gross nitrification rate more significantly than the gross ammonification rate. The gross nitrification ceased until soil water content reached 60%WHC, indicating that soil water availability between 60% and 100% WHC was not a limiting factor in the nitrification process for the shrubland soils in this study. The ammonium (NH_4_^+^) immobilization was significantly lower than nitrification irrespective of varying environmental conditions, even though the NH_4_^+^ consumption rate might be overestimated, uncovering two putative processes: (1) heterotrophic nitrification process; (2) and more competitive nitrifying bacteria than NH_4_^+^-immobilizing microorganisms. Our study is indispensable for assessing the stability and sustainability of soil N cycling in the shrub ecosystem under climate changes.

## 1. Introduction

Nitrogen (N) is the most abundant element in the atmosphere and is also the key factor in limiting the productivity of terrestrial ecosystems. As far as we know, more than 90% of N in soil exists in the form of macromolecular organic N [1]. While some plants utilize small-molecule soluble organic N to fulfill their anabolic needs (DON) [2,3], most plants tend to absorb and utilize inorganic N [4,5]. Plant growth largely depends on the supply of inorganic N, which is closely related to various soil N transformation processes in terrestrial ecosystems. The processes of soil N transformation not only determine the content of inorganic N available to plants in the soil, thereby affecting the growth, development and productivity of the ecosystem, but also determine the distribution of main inorganic N forms, affecting the potential and pathways of N loss in the soils [5,6].

Soil N transformation rates are classified as net and gross N transformation rates. Net N transformation rates are measured by the changes in the sizes of soil inorganic-N pool per unit time [7]. Gross N transformation rates refer to the actual amount of inorganic N provided or consumed by microorganisms in the soil through specific microbial processes per unit time [8]. Net ammonification and net nitrification rates are important indicators to reflect the basic status of inorganic N available to plants in the soil of ecosystems. However, the net N transformation rate is the sum of a series of inorganic N production and consumption processes, which can only show the net input (net output) rather than the actual total amount of each N transformation process [9,10]. The development of isotope technology has led to an inevitable trend toward quantitative studies of gross N transformations, showing the complete dynamics of soil N cycling.

Soil gross N transformations are affected by various factors, among which soil temperature and water content are two of the most important factors. Climate change often causes variations in soil temperature and moisture in ecosystems. The effects of soil temperature on soil N transformation are mainly caused by the temperature sensitivity of soil microbial activities. As soil temperature increases smoothly, microbial growth and activity increase, thereby increasing gross N transformation rates [9,11]. Wang et al. [12] showed that the rates of soil gross mineralization and NH_4_^+^ immobilization increased with the increment of temperature in the range of 5–35 °C. Lang et al. [13] conducted laboratory incubation studies on two different types of soil, and pointed out that the increase in temperature could promote the nitrification and the immobilization of inorganic N by microorganisms. However, the effect of temperature on soil N transformation rate is not always straightforward and is ecosystem-specific due to the indigenous microbial diversities under different climate and environmental conditions. Even for the same soil, the rates of N transformations may show inconsistent responses to temperature variations in different temperature ranges [11,12]. Soil water can be used as a carrier of the microbial substrate and can also affect the diffusion of oxygen in the soil, thus affecting the rates of biological N transformations [14]. Soil gross N mineralization rates could be enhanced by increasing soil water content [15,16]. Some studies have pointed out that the rate of soil gross nitrification was directly proportional to soil moisture content [14,17]. However, gross N nitrification rates showed a downward trend when the soil water content exceeded a threshold [18,19]. Therefore, the effects of soil temperature and moisture on N transformation processes can provide a theoretical basis for further understanding the responses of the soil N cycling to climate change.

Shrubland is an important terrestrial ecosystem in China, with a total area of 743,000 km^2^, accounting for one-fifth of China′s land area, of which 288,000 km^2^ is located in the temperate zone of northern China. North China is one of the main distribution areas of shrubland [20]. In recent years, due to the degradation of some native vegetation and the restoration or reconstruction of some native shrubs, the distribution range of shrub vegetation in China is expanding rapidly [21]. With the increment in area, the influence of shrubland on the function and nutrient cycle of the terrestrial ecosystem in China extensively enhanced [20,21]. Shrubland is a N-poor ecosystem, and N transformation processes in soil play a crucial role in maintaining the productivity of the ecosystem [20,22,23]. In addition, as a natural or semi-natural ecosystem, shrubland is normally considered a relatively sensitive ecosystem which is easily affected by environmental changes and human activities [24,25]. There are few studies on soil N transformation in shrubland ecosystems worldwide, focusing mainly on the effects of fire and N deposition on soil N transformation rates [26,27,28]. These studies indicated that fire disturbance could lead to an increase in soil gross mineralization and nitrification rates in shrubland [27,28]. Moreover, changes in soil N transformation characteristics may cause alterations in the distribution of inorganic N forms in soil, resulting in changes in N loss potentials and pathways [29]. In China, however, soil N transformation in shrub ecosystems was poorly understood. To our knowledge, only one report has demonstrated that the soil gross mineralization and nitrification rates of shrubland in karst areas in southwest China were slightly higher than those in grassland and significantly lower than those in forest [30]. Taken together, the overall understanding of the characteristics of soil N transformation and soil N cycle in shrubland ecosystems is still deficient.

Changes in temperature and precipitation caused by climate change will lead to disturbance in soil hydrothermal conditions, which greatly affects the soil N cycling. This study aimed to investigate the impact of temperature and humidity changes on soil N transformation in a typical shrubland in North China as part of efforts to better comprehend the mechanisms of climate change on soil N cycling. In the current study, we measured the rates of gross N ammonification, nitrification, and inorganic N immobilization in soils at different depths (0–10 cm and 10–20 cm) under varying temperatures and humidity conditions in a typical North China mountain shrub ecosystem in the Yanshan mountain and hilly region. The objectives of this study are: (1) to explore the effects of soil temperature on gross N transformation rates in shrubland; (2) to evaluate the responses of soil gross N transformation rates to soil water conditions in shrubland; (3) to compare whether there are differences in gross N transformation rates of soils at different depths (0–10 cm vs. 10–20 cm).

To this end, we hypothesize that: (1) gross ammonification, nitrification, and NH_4_^+^ immobilization rates would be significantly increased by increasing soil temperature; (2) ammonification and nitrification would be facilitated by higher soil moisture levels; (3) the rates of gross N transformations in the soil would decrease with an increase in soil depth.

## 2. Materials and Methods

### 2.1. Study Site Description

**Figure 1:**
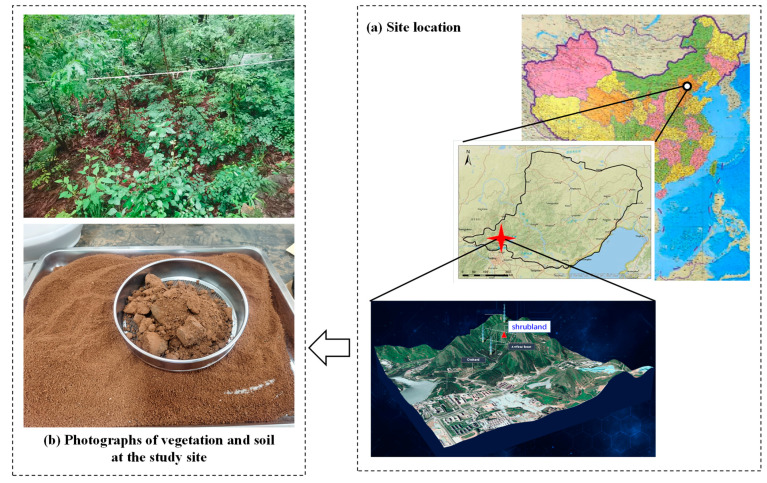
The location, biotope and soil of the study site.

The soil sampling site (40°25′0.84″ N, 116°39′14.76″ E; altitude: 140 m) is located in a shrub plot of Beijing Yanshan Earth Critical Zone National Research Station in Huairou District, Beijing, China (Figure 1). The vegetation in the sampling site is dominated by the *Vitex negundo* var. *heterophylla* (Franch.) Rehd., *Grewia biloba* G. Don, *Rhamnus schneideri* Levl., all of which are typical shrubs in North China. The open space between shrubs is covered by herbs such as *Arthraxon hispidus* (Trin.) Makino and *Deyeuxia pyramidalis* (Host) Veldkamp, and vines such as *Dioscorea nipponica* Makino, *Vitis amurensis* Rupr., and *Celastrus orbiculatus* Thunb. The study area is described as having a warm temperate semi-humid climate. The mean annual air temperature was 12.4 °C (2020–2021), with maximum and minimum monthly average air temperatures of 37.1 °C in June and −18.3 °C in January, respectively. In 2021, the annual precipitation in urban Beijing was 924 mm, the highest since 1978 (the mean annual precipitation from 1978 to 2020 was 544.3 mm). The annual precipitation in Huairou was 1032.6 mm in 2021, of which approximately 88% (906.8 mm) occurred from June to September. In 2022, precipitation in Huairou from June to September was 456 mm, nearly less than half compared to the same period in 2021.The soil is a Chromic Cambisol according to WRB 2022 [31], with a pH of 7.4 ± 0.4 (mean ± standard error, 0–10 cm).

### 2.2. Soil Sampling and Experimental Design

The soil was divided into two depths of 0–10 cm (SL10) and 10–20 cm (SL20), respectively, in this study. Three plots were randomly selected for soil sampling. Soil samples of 0–10 cm depth and 10–20 cm depth were collected from the mineral layer soil in July 2022. After removing the litter, two composite soil samples of SL10 and SL20 were collected from each plot. All the soils were sieved using a 2 mm-mesh, and stones, leaves, and fine roots were manually removed. The soil samples at two depths were divided into two subsamples, one of which was stored at 4 °C for incubation experiments, while the other subsample was air-dried for the detection of soil physical and chemical properties.

In order to study the effects of soil temperature and humidity on soil gross N transformation rates, four temperature control experiments and three humidity experiments were set up in this study. The incubation temperature was set to 5, 15, 25, and 35 °C, respectively. The soil water content in the incubation experiment was adjusted to 30%, 60%, and 100% water-holding capacity (WHC), respectively.

### 2.3. Measurements of Gross N Transformation Rates

The ^15^N isotope pool dilution method was used to measure the rates of soil gross N transformation in laboratory incubation. For each depth of soil (SL10 and SL20), two groups of 250 mL conical flasks were prepared, each containing 30 g of fresh soil. One group of soils was homogenously labeled with a 30 at% ^15^N-enriched ammonium sulfate ((^15^NH_4_)_2_SO_4_) solution, and the other with a 30 at% ^15^N-enriched potassium nitrate (K^15^NO_3_) solution. 1 mL of labeling solution was added into each flask, causing an increase of 1 mg N kg^−1^ soil in ambient soil inorganic N content. Three replicates (2 flasks for each replicate) were set for each incubating temperature and humidity condition. After labeling, parts of the soil were adjusted to 60% WHC, sealed with parafilm, and subsequently incubated in the dark at 5, 15, 25, and 35 °C, respectively. The other parts of the soil were adjusted to 30%, 60%, and 100% WHC, respectively, sealed with parafilm, and then incubated in the dark at 25 °C. A 24 h-pre-incubation was performed to provide an equilibration of N and microorganisms in the labeled soil. The soil samples were extracted with 75 mL 1 M KCl solution at 24 (*t*_1_) and 48 h (*t*_2_) after labeling. Soil extracts were divided into two parts for further processing. 30 mL extracts were adjusted to be alkaline by adding MgO for the diffusion process. The NH_4_^+^ from ^15^NH_4_^+^-labeled soil extracts was trapped through the acid filter papers, according to the method described by Dannenmann et al. [32]. For ^15^NO_3_^−^-labeled soil extracts, NH_4_^+^ should be removed by shaking and acid absorption, and then NO_3_^−^ was trapped after being reduced to NH_4_^+^ by Devarda’s alloy. The remaining extracts (about 20 mL each) were filtered with 0.22 μm syringe filters and then frozen at −20 °C for determination of NH_4_^+^ and NO_3_^−^ concentrations using a continuous flow analyzer (Seal AutoAnalyzer AA3, Norderstedt, Germany). The filter papers harvested during the diffusion process were dried and further prepared for the analysis of ^15^N enrichment by a stable isotope ratio mass spectrometer (Isoprime100, Elementar, Hanau, Germany).

Gross rates of ammonification (GA), nitrification (GN), and NH_4_^+^ consumption (CA) were calculated according to the formulas proposed by Kirkham and Bartholomew [33]:

(1) GA or GN=M0-M1t×log⁡(H0M1H1M0)log⁡(M0M1)

(2) CA=M0-M1t×log⁡(H0H1)log⁡(M0M1)

where *M*_0_ and *M*_1_ represent the concentration of ^14+15^NH_4_^+^ (for GA and CA) or ^14+15^NO_3_^−^ (for GN) in soils at *t*_1_ and *t*_2_, respectively; *H*_0_ and *H*_1_ indicate the concentration of ^15^NH_4_^+^ (for GA and CA) or ^15^NO_3_^−^ (for GN) in soils at *t*_1_ and *t*_2_, respectively; *t* is the incubation time.

We calculated gross NH_4_^+^ immobilization (IA) by subtracting gross nitrification from NH_4_^+^ consumption [34].

### 2.4. Measurements of Soil Properties

In addition to the measurement of gross N transformation rates, part of the soil sample was sieved through a 2 mm-mesh and then extracted with 1 M KCl solution and deionized water (soil/solution ratio was 1:5) for the determination of NH_4_^+^, NO_3_^−^, and water-soluble organic carbon (WSOC) concentrations, respectively. The filtered extracts were immediately frozen at −20 °C for analysis on a continuous flow analyzer (Seal AutoAnalyzer AA3, Norderstedt, Germany). Soil gravimetric water content (SWC) was measured by oven-drying soil samples at 105 °C to constant weight. The soil texture (<1 mm) was measured by a laser particle size analyzer (Malvern Mastersizer 2000, Malvern, UK). Soil organic carbon (SOC) content was determined by the method of potassium dichromate oxidation–external heating. Soil total N content was determined by the Kjeldahl method. The meteorological data mainly come from the dataset of the Beijing Meteorological Bureau and Beijing Statistical Yearbook.

### 2.5. Data Processing and Statistical Analysis Methods

SPSS22.0 Statistics software package (IBM SPSS Statistics) was used for statistical analysis and the Origin 2018 software package (OriginLab Ltd., Northampton, MA, USA) was used for data analyzing and drawing. The *t*-test of independent samples was applied to conduct a statistical significance test for the differences in soil properties, inorganic N concentrations, and gross N turnover rates among soils at different depths, as well as the differences in gross N turnover rates under different incubation temperatures and moisture contents, and the differences in the variation of ^15^N enrichment and inorganic N concentration during incubation. Simple exponential regression analyses were performed to describe the correlation between soil temperature and gross ammonification rates.

## 3. Results

### 3.1. Soil Properties

Soil pH and texture did not vary greatly (Table 1). The contents of soil organic carbon (SOC) and total N (TN) in SL10 were significantly higher than those in SL20 (*p* < 0.001, Table 1), while there was no significant difference in the C/N ratio (averaged 10.6 for SL10 and 10.1 for SL20). SL10 had significantly greater concentrations of NH_4_^+^ and NO_3_^−^ than SL20 (*p* < 0.001). Similarly, the WSOC concentration of SL10 was significantly higher than that of SL20 (*p* < 0.01).

### 3.2. Changes of ^15^N Enrichments and Soil Inorganic N Concentrations during the Incubation Period

In the ^15^NH_4_^+^ labeled soils, the ^15^N atom% excess of the NH_4_^+^ pool reduced during the incubation period (from *t*_1_ to *t*_2_) at all incubating temperatures and humidity conditions (Table 2), suggesting that NH_4_^+^ produced by ammonification entered the labeled NH_4_^+^ pool in its natural abundance. Throughout the incubation period from *t*_1_ to *t*_2_, the ^15^N atom% excess of the NO_3_^−^ pool in the ^15^NO_3_^−^ labeled soils exhibited downward trends under all incubating temperature and humidity conditions (Table 2), indicating that NO_3_^−^ with the natural abundance produced by nitrification entered the labeled NO_3_^−^ pool and had a dilution effect on ^15^N. In the ^15^NH_4_^+^ labeled soil, the NH_4_^+^ concentration decreased significantly at all incubating temperature and humidity conditions from *t*_1_ to *t*_2_, except for a small increase at 5 °C for SL10 (Table 2). In the ^15^NO_3_^−^ labeled soil, in contrast, the NO_3_^−^ concentration increased significantly at all incubating temperature and humidity conditions from *t*_1_ to *t*_2_, except for SL20, which increased, but not significantly, at 5 °C (Table 2).

### 3.3. Gross N Transformation Rates under Different Incubation Temperatures

**Figure 2:**
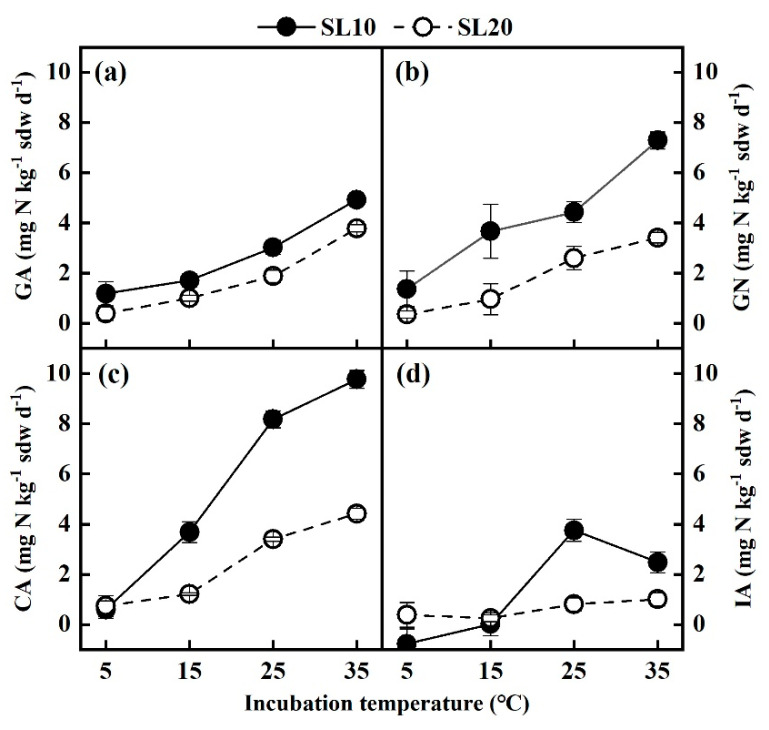
Gross rates of N transformations under different incubation temperatures. (**a**) Gross ammonification rates (GA), (**b**) gross nitrification rates (GN), (**c**) gross rates of NH_4_^+^ consumption (CA), (**d**) gross rates of NH_4_^+^ immobilization (IA).

The gross rates of ammonification (GA) and nitrification (GN) were both higher in SL10 soils than those in SL20 soils at all incubation temperatures (Figure 2a,b). The differences were significant at 15, 25, and 35 °C (*p* = 0.006, 0.006, and 0.002, respectively), but not significant at 5 °C. Both gross rates of ammonification and nitrification were sensitive to temperature changes. The GA of SL10 and SL20 increased exponentially with incubation temperature (SL10: *R*^2^ = 0.993, *p* = 0.002; SL20: *R*^2^ = 0.992, *p* = 0.003, Figure 2a), increasing from 1.2 ± 0.5 and 0.4 ± 0.3 mg N kg^−1^ sdw d^−1^ to 4.9 ± 0.2 and 3.8 ± 0.1 mg N kg^−1^ sdw d^−1^, respectively. The temperature sensitivity coefficient of GA, indicated by *Q*_10_ value, was 1.6 and 2.1, for SL10 and SL20, respectively. In the SL10 soil, the GN increased more pronounced between 5 to 15 °C and 25 to 35 °C than between 15 to 25 °C, yet in the SL20 soil, the GN increased more pronounced between 15 to 25 °C than between 5 to 15 °C and 25 to 35 °C (Figure 2b). The GN of SL10 and SL20 grew from 1.4 ± 0.7 and 0.4 ± 0.1 mg N kg^−1^ sdw d^−1^ at 5 °C to 7.3± 0.3 and 3.4 ± 0.2 mg N kg^−1^ sdw d^−1^ at 35 °C, respectively. In SL10 soil, GN exceeded GA at all incubation temperatures, and the difference was significant at all incubation temperatures except 5 °C (Figure 2a,b). In contrast, there was no significant difference between GN and GA in SL20 soil at all incubation temperatures (Figure 2a,b).

The gross rates of NH_4_^+^ consumption (CA) in SL10 soils were higher than that in SL20 soils at all incubation temperatures (Figure 2c). The differences at 15, 25, and 35 °C were more significant (*p* = 0.019, 0.007, and 0.001, respectively) than at 5 °C. Similar to GA and GN, the CA of SL10 and SL20 also increased with the increase of incubation temperature from 5 to 35 °C. In the SL10 soil, the CA increased from 0.6 ± 0.4 to 9.8 ± 0.4 mg N kg^−1^ sdw d^−1^ as the incubation temperature increased from 5 to 35 °C. There was a linear positive correlation between CA and GN in the SL20 soil between 5 and 35 °C (*R*^2^ = 0.99, *p* = 0.001), with CA being approximately 1.3 times GN. The gross rate of NH_4_^+^ immobilization (IA) of SL10 at 25 and 35 °C was higher than that at 5 and 15 °C, but the temperature dependence was not observed for SL20 soils. Notably, the IA calculated by subtracting the GN from CA exhibited a negative value in SL20 soil at 5 °C.

### 3.4. Gross N Transformation Rates under Different Soil Moisture Conditions

**Figure 3:**
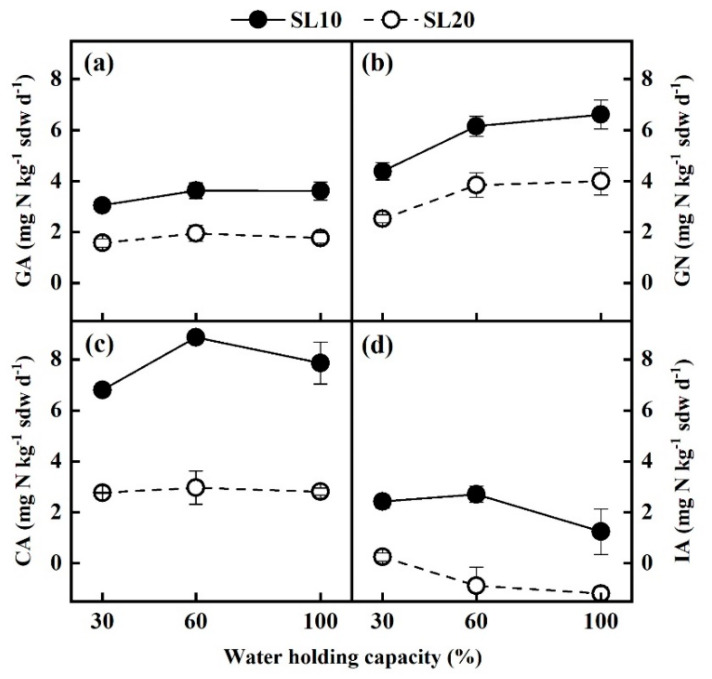
Gross rates of N transformations under different soil water contents during the incubation period. (**a**) Gross ammonification rates (GA), (**b**) gross nitrification rates (GN), (**c**) gross rates of NH_4_^+^ consumption (CA), (**d**) gross rates of NH_4_^+^ immobilization (IA).

The GA, GN, and CA in SL10 soil were consistently significantly higher than those in SL20 soil, irrespective of incubation soil moisture (*p* < 0.01, Figure 3a–c). In the SL10 soil, the GA increased slightly as the moisture increased from 30% to 60% WHC (Figure 3a), as compared to more significantly for CA (Figure 3c). However, with the further increase of soil moisture, GA and CA leveled off (Figure 3a,c). For SL20 soil, GA and CA showed no significant difference under different soil moisture contents (Figure 3a,c). The GN of SL10 and SL20 at 60% and 100% WHC was significantly higher than that at 30% WHC (*p* < 0.05), but there was no significant difference between 100% and 60% WHC (Figure 3b). In SL10 and SL20 soils, GN was significantly higher than GA under all incubation humidity conditions (*p* < 0.01, Figure 3a,b). Notably, the IA calculated by subtracting the GN from CA was negative in SL20 with soil moisture of 60% and 100% WHC (Figure 3d). There was no significant difference in IA in SL10 soil under distinct soil moisture incubations (Figure 3d).

## 4. Discussion

### 4.1. Errors of NH_4_^+^ Immobilization Rates and a Possibility of Heterotrophic Nitrification

In all the ^15^N dilution experiments under current conditions, three calculated NH_4_^+^ immobilization rates were negative (Figure 2d and Figure 3d), accounting for ~21% of all the data. Similar cases have been reported in previous studies [32,35], but it is theoretically impossible to have negative NH_4_^+^ immobilization rates. Dannenmann et al. [32] attributed the negative values mainly to the spatial variability of background inorganic N concentration in soil and the nonuniform distribution of the ^15^N label. As such, from an ecological perspective, the calculated negative rates should be set as 0. In this study, we proposed a secondary possibility. The gross NH_4_^+^ immobilization rate was estimated by subtracting the gross nitrification rate from gross NH_4_^+^ consumption rate. When the gross nitrification rate exceeded the NH_4_^+^ consumption rate, the NH_4_^+^ immobilization rate became negative. In this study, the gross nitrification rate indeed represented the rate of NO_3_^−^ production in soil. If the soil nitrification process consumed organic N in addition to NH_4_^+^, that is, in the presence of heterotrophic nitrification, the N consumed in the nitrification process, including heterotrophic and autotrophic nitrification, may surpass the total consumption of NH_4_^+^. However, it is impossible to distinguish the N consumed by heterotrophic nitrification in the calculation process of the ^15^N pool dilution method. Therefore, the decrement in the calculation may be higher than the N consumed by the actual autotrophic nitrification, resulting in the underestimation of NH_4_^+^ immobilization and even a negative value.

Our findings disclosed that where the NH_4_^+^ immobilization rate was negative, the gross nitrification rate was greater than the gross ammonification rate. Around 79% of gross nitrification rates were higher than gross ammonification rates. Similar results were reported in a semi-arid steppe in Inner Mongolia and an alpine meadow in southern Germany [10,36]. All the above studies speculated that this was caused by heterotrophic nitrification occupying a significant proportion of the nitrification process. Although autotrophic nitrification was considered predominant for NH_4_^+^ consumption in many soils [5], studies have shown that heterotrophic nitrification plays a critical role [37,38,39,40]. The ^15^N pool dilution technique used in this study was unable to differentiate heterotrophic nitrification and autotrophic nitrification, which prevented an accurate assessment of heterotrophic nitrification’s contribution to shrub soil N cycling.

### 4.2. Effect of Temperature on Soil Gross N Transformation Rates

Soil N transformations are microbial-mediated processes which are greatly driven by soil temperature [41]. The microorganisms involved in the N transformation include *Nitrososphaera viennensis*, *Nitrosocosmicus franklandus*, *Nitrosomonas communis*, *Candidatus Nitrosotalea devanaterra*, etc. [42,43]. In this study, the results showed that gross ammonification rates increased exponentially as the incubation temperature increased from 5 to 35 °C in SL10 and SL20 soils (*R*^2^ > 0.99, *p* < 0.01, Figure 2a), which was consistent with ^15^N tracing studies in subtropical broad-leaved forest and coniferous forest soils [12,44,45]. In short, ammonification in soil is composed of two processes: decomposition of labile organic N and decomposition of recalcitrant organic N [45]. The decomposition of recalcitrant organic N is less temperature-sensitive than that of labile organic N in subtropical forests, so it can only be stimulated at higher temperatures [44]. Furthermore, the non-synchronous response of the decomposition of recalcitrant organic N and labile organic N to the change in temperature would have resulted in a non-linear, i.e., an exponential increase of the gross ammonification rate with temperature [45].

Nitrification has been shown to be more temperature-sensitive than mineralization and immobilization [9,11]. Consistent results were observed in the SL10 soil of the current study (Figure 3a,b,d). However, nitrification was only more sensitive to temperature change than immobilization for SL20, not for ammonification. This may be related to the fact that NH_4_^+^ and WSOC of SL20 were significantly lower than those of SL10 (*p* < 0.01, Table 1) since the temperature sensitivity of nitrification may be reduced when available C and NH_4_^+^ are limiting factors [44]. Inconsistently, some studies unraveled that the nitrification rate was not sensitive to temperature changes. In another scenario, the nitrification rate increased at a certain temperature range (from 0 to 25 °C) but decreased afterward [12,46]. Accordingly, the effect of temperature on soil N transformation rate is of high variability and ecosystem-specific due to differences in climate and environmental conditions [11,12]. Since microorganisms dominate most processes of the soil nitrogen cycle, including ammonification, nitrification, and inorganic N immobilization, our results also indicated that the associated soil microbial community structure may be altered under the influence of long-term climate change.

In recent years, global radiative forcing has been increasing, causing the climate system to be continuously heating up. The global land surface temperature in 2011–2020 was 1.59 °C higher than that in 1850–1900. Almost all land regions have experienced extreme heat in recent years [47]. Given the current climate change scenario, the increase of soil temperature is inevitable. Soil temperature in North China is generally much lower than 35 °C [48,49]. According to the results of this study, the rates of gross ammonification, nitrification, and NH_4_^+^ consumption will be significantly accelerated as a result of climate change, causing the rise of the soil temperature in the shrub ecosystem of North China. Nitrification is one of the main processes for the production of N_2_O, an important greenhouse gas in the atmosphere [50]. Its enhancement will lead to the increase of soil N_2_O emission, which will have negative feedback on climate change. In addition, the nitrification process dominated NH_4_^+^ consumption, and the rate of NH_4_^+^ consumption tended to exceed the rate of ammonification. Consequently, the NO_3_^−^ concentration in soils will increase, and the NH_4_^+^ availability for plants may be weakened, which is not conducive to the growth of most plants and would decline the primary productivity of this shrub ecosystem. When nitrate becomes the predominant form of N in soils, the risk of N loss will be promoted [51]. On one hand, the possibility of NO_3_^−^ leaching increases with precipitation and runoff; on the other hand, denitrification consumes NO_3_^−^ and releases N_2_O, N_2_ and other gases into the atmosphere, resulting in N loss [52]. In summary, changes in soil N transformation due to rising temperature may be detrimental to the sustainability of this shrub ecosystem and may enhance climate warming.

### 4.3. Effect of Soil Moisture on Gross N Transformation Rates

The variations of moisture strongly affect the activity of microorganisms, the concentration of oxygen, and the transportation of nutrients in soils, thus impacting soil N transformation processes [14,53]. The soil gross N mineralization rates could be enhanced by increasing soil water content [15,16]. According to a study in acid subtropical forest soil, mineralization at 50%, 70%, and 90% WHC was higher than that at 30% WHC in broad-leaved forest soil, and mineralization at 70% and 90% WHC was higher than that at 30% and 50% WHC in coniferous forest soil [14]. Hu et al. [19] discovered a linear positive correlation between mineralization rate and soil water content in an alpine shrub meadow ecosystem. However, some studies have shown that the gross rate of ammonification was not affected by changes in soil water content [40,54]. Likewise, in the current study, changes in soil moisture content did not inspire a significant variation in the gross ammonification rate, except for a slight increase (18.9%) in gross ammonification in SL10 when soil moisture increased from 30% to 60% WHC. It was indicated that soil moisture was not a limiting factor in ammonification in the shrub soils.

The gross rates of nitrification were significantly higher at 60% and 100% WHC than that at 30% WHC in both SL10 and SL20 soils, consistent with previous studies in subtropical forest soils and alpine soils [14,55]. In principle, soil nitrification generally increases first and then decreases as soil moisture content increases [19,56,57]. This finding is associated with soil microhabitats under different moisture conditions. A moderate increase in soil moisture content would likely stimulate microbial activity and enhance substrate diffusion, thus promoting the nitrification rate. Excessive soil moisture, however, can create an anaerobic environment, inhibiting the activities of nitrifiers and promoting denitrifiers to become active, weakening nitrification. [18,54]. An increase in soil moisture content from 60% to 100% WHC did not significantly affect gross nitrification rates in this study, indicating that soil water availability between 60% and 100% WHC did not limit nitrification for shrubland soils This result highlights that for this shrub soil, soil water content ranging from 60% to 100% WHC could be considered the optimal humidity conditions for nitrification. Comparably, the responses of gross nitrification rates to soil moisture changes are collective effects of soil type, soil bulk density, and soil porosity [54]. For instance, Linn et al. [58] showed that 60% WFPS (soil water-filled porosity) was the optimal soil water content for nitrification in tillage system soils. Kiese et al. [59] found that 65% WFPS was the optimal soil moisture condition for nitrification in a tropical rainforest soil in Australia. Cheng et al. [14] reported that the soil moisture most conducive to nitrification in soil of a subtropical forest was 70–90% WHC. 

Precipitation extremes have intensified as a result of climate change and will continue to do so [60,61]. In recent years, Beijing has experienced frequent extreme precipitation and heavy precipitation in summer. As a result, nitrification will be stimulated by the abrupt increase of soil moisture content, resulting in the release of N_2_O, NO and NO_2_ into the atmosphere, as well as the production of NO_3_^−^ that is easy to be eluviated, which harms air quality and increases the risk of N loss in soils. If precipitation extremes result in the saturation of soil moisture, nitrification may be reduced. However, higher nitrification and less NO_3_^−^ consumption would accumulate the soil NO_3_^−^ before precipitation occurs. In this case, the denitrification would likely be accelerated, thus increasing the emission of N_2_O and N_2_, which is one of the pivotal pathways for soil N loss. Therefore, under the condition of extreme precipitation caused by climate change, these shrub soils in North China will face the risk of aggravating N loss, and will likely emit more N_2_O to the atmosphere, thus contributing to the intensification of the climate-warming process.

### 4.4. Effect of Soil Depth on Gross N Transformation Rates

In the current study, the gross rates of ammonification, nitrification, and NH_4_^+^ consumption in SL10 soil were higher than those in SL20 soil, irrespective of incubation temperatures and soil moisture (Figure 2 and Figure 3). Similar results were also presented by Sharma et al. [62], showing that in tropical montane soils of the Himalayas there was a positive correlation between gross ammonification and nitrification rate and SOC and TN, and the availability of N controlled ammonification and nitrification. Similarly, the contents of SOC and TN in SL10 soil were significantly higher than those in SL20 soil of the shrubland ecosystem in this study (*p* < 0.001, Table 1), which was consistent with ammonification and nitrification patterns, underlying the importance of SOC in driving N cycling in shrubland. Increases in gross mineralization were accompanied by increases in total carbon (TC) and TN concentrations in some soils, demonstrating that soil organic matter content is a major factor affecting soil N transformation [63,64,65,66]. Soil TN content had an indirect effect on soil mineralization by directly affecting microbial biomass [65,67], i.e., soil with a higher TN content promoted gross ammonification by increasing microbial biomass. Soil organic N content affected the soil mineralization process, while SOC content played a vital role in regulating the microbial immobilization process. In soils with depleted SOC, heterotrophic microorganisms would be restricted, and nitrifiers would be more competitive for NH_4_^+^, which improved nitrification [68]. In general, gross ammonification in soil would be inhibited by increasing the soil C/N ratio, although microbial immobilization would be promoted [7,69,70]. Gross ammonification was improved, while immobilization was impaired when the C/N ratio of soil organic matter was less than 18 [67,71]. The C/N ratio of shrubland soil in this study was between 10 and 11, which could indirectly interpret the relatively low rate of NH_4_^+^ immobilization.

The processes of N transformation in soils occur interchangeably. Some previous studies have shown that the rates of gross nitrification and NH_4_^+^ immobilization usually increased with the rate of gross ammonification [19,65]. It was attributed to the fact that NH_4_^+^, the product of ammonification, is an important substrate for both autotrophic nitrification and microbial immobilization [5,52]. When sufficient NH_4_^+^ was provided by the ammonification process, it was possible to meet the substrate requirements for both nitrification and NH_4_^+^ immobilization. In this study, the depth-dependent gross nitrification rate change was basically in line with the gross ammonification rate, indicating that the nitrification would have been greatly influenced by the ammonification in the shrubland soils. However, the gross rate of NH_4_^+^ immobilization was almost invariably at a relatively low level. In addition to the error caused by the calculation method, it also indicated that the immobilization of NH_4_^+^ by heterotrophic microorganisms could not outcompete with nitrification, putatively being affected by the relatively low C/N ratio (<18).

## 5. Conclusions

Our study focused on the response of soil gross N transformation rates to the changes in temperature and moisture content in a typical shrub ecosystem in the Yanshan mountain and hilly region. The results showed that gross ammonification rates increased exponentially as the incubation temperature increased from 5 to 35 °C, and the gross nitrification rate was also significantly stimulated with the increase in incubation temperature. The temperature-sensitive N transformation in such shrub soils should be eye-catching due to the ongoing climate changes worldwide, which would cause a previously overlooked risk of N loss. The effect of soil moisture on the nitrification process of shrubland soil was dependent upon the soil water contents before the change of moisture. The increase of soil moisture from 30% WHC to 60% and 100% WHC would significantly accelerate nitrification, resulting in an increased possibility of NO_3_^−^ leaching, while the increase of soil moisture from 60% to 100% WHC had no significant effect on nitrification, suggesting that soil moisture was not a limiting factor for nitrification in this study. Due to the negative NH_4_^+^ immobilization rates, and the greater gross nitrification rate over gross ammonification and NH_4_^+^ consumption rate, we speculated that a considerable proportion of heterotrophic nitrification processes might occur in the soils of this shrub ecosystem. In summary, our results suggested that under the background of climate change altering soil temperature and moisture, the N transformation processes of the shrub soils in North China would be disturbed, leading to an increased risk of soil N loss. Our study is indispensable for assessing the stability and sustainability of soil N cycling in the shrub ecosystem under climate changes.

## Figures and Tables

**Table 1:** The main physical and chemical properties of the studied soils.

Soil Depth	SL10	SL20
WHC (%)	53.1 ± 1.5 ^a^	49.6 ± 1.5 ^a^
pH	7.4 ± 0.4 ^a^	7.5 ± 0.2 ^a^
Clay (%)	6.8 ± 1.1 ^a^	7.6 ± 1.0 ^a^
Silt (%)	28.1 ± 4.0 ^a^	30.3 ± 4.4 ^a^
Sand (%)	65.1 ± 3.7 ^a^	62.2 ± 4.5 ^a^
SOC (g kg^−1^)	19.1 ± 1.5 ^a^	14.0 ± 1.3 ^b^
TN (g kg^−1^)	1.81 ± 0.09 ^a^	1.38 ± 0.08 ^b^
WSOC (mg C kg^−1^ sdw)	11.7 ± 0.9 ^a^	9.1 ± 0.3 ^b^
NH_4_^+^ (mg N kg^−1^ sdw)	11.1 ± 0.8 ^a^	6.0 ± 0.7 ^b^
NO_3_^−^ (mg N kg^−1^ sdw)	10.9 ± 0.7 ^a^	6.7 ± 0.3 ^b^

SL10: Soil with a depth of 0–10 cm; SL20: Soil with a depth of 10–20 cm; WHC: Water-holding capacity; SOC: Soil organic carbon; TN: Total nitrogen; WSOC: Water soluble organic carbon; sdw: soil dry weight. The values after the “±” represent the standard errors of 3 or more replicates. The superscripts of different lowercase letters (a and b) indicate significant differences between different depths (*p* < 0.01).

**Table 2:** The ^15^N enrichments and inorganic N concentrations were measured at time *t*_1_ and *t*_2_ during incubation experiments.

	Incubation Temperature/Moisture	SL10		SL20	
		*t*_1_	*t*_2_	*t*_1_	*t*_2_
^15^N enrichment of NH_4_^+^ in the ^15^NH_4_^+^ labeled soils (atom%)	5 °C	2.35 ± 0.02	2.18 ± 0.08	2.90 ± 0.07	2.79 ± 0.02
	15 °C	2.13 ± 0.03	1.88 ± 0.004	2.66 ± 0.04	2.39 ± 0.004
	25 °C	1.81 ± 0.02	1.31 ± 0.04	2.21 ± 0.02	1.77 ± 0.03
	35 °C	1.35 ± 0.02	0.92 ± 0.004	1.70 ± 0.01	1.21 ± 0.01
	30% WHC	1.65 ± 0.04	1.30 ± 01	2.09 ± 0.01	1.76 ± 0.02
	60% WHC	1.59 ± 0.02	1.17 ± 0.02	2.10 ± 0.03	1.68 ± 0.03
	100% WHC	1.60 ± 0.08	1.02 ± 0.04	2.05 ± 0.05	1.60 ± 0.02
^15^N enrichment of NO_3_^−^ in the ^15^NO_3_^−^ labeled soils (atom%)	5 °C	2.54 ± 0.01	2.34 ± 0.11	3.91 ± 0.06	3.77 ± 0.11
	15 °C	2.41 ± 0.06	2.02 ± 0.06	3.46 ± 0.05	3.18 ± 0.12
	25 °C	2.05 ± 0.01	1.76 ± 0.02	3.27 ± 0.03	2.72 ± 0.06
	35 °C	1.89 ± 0.04	1.52 ± 0.02	3.02 ± 0.01	2.42 ± 0.03
	30% WHC	2.04 ± 0.02	1.73 ± 0.03	2.09 ± 0.01	1.76 ± 0.02
	60% WHC	1.96 ± 0.02	1.61 ± 0.005	2.10 ± 0.03	1.68 ± 0.03
	100% WHC	1.95 ± 0.02	1.59 ± 0.04	2.05 ± 0.05	1.60 ± 0.02
NH_4_^+^ concentrations in the ^15^NH_4_^+^ labeled soils (mg N kg^−1^ sdw)	5 °C	13.0 ± 0.2	13.6 ± 0.2	8.7 ± 0.04	8.3 ± 0.1
	15 °C	12.3 ± 0.2	10.3 ± 0.1	8.1 ± 0.1	7.8 ± 0.1
	25 °C	10.0 ± 0.03	4.9 ± 0.1	7.7 ± 0.1	6.2 ± 0.2
	35 °C	11.1 ± 0.4	6.3 ± 0.2	8.6 ± 0.2	7.9 ± 0.2
	30% WHC	11.6 ± 0.2	7.8 ± 0.3	8.1 ± 0.1	6.9 ± 0.2
	60% WHC	11.4 ± 0.3	6.2 ± 0.02	7.4 ± 0.1	6.4 ± 0.7
	100% WHC	8.1 ± 0.5	3.8 ± 0.1	6.3 ± 0.3	5.2 ± 0.1
NO_3_^−^ concentrations in the ^15^NO_3_^−^ labeled soils (mg N kg^−1^ sdw)	5 °C	12.3 ± 0.1	12.2 ± 0.4	7.2 ± 0.02	7.8 ± 0.1
	15 °C	11.0 ± 0.4	9.3 ± 0.1	7.3 ± 0.1	7.0 ± 0.2
	25 °C	9.0 ± 0.1	4.0 ± 0.04	7.0 ± 0.1	5.4 ± 0.2
	35 °C	10.3 ± 0.3	5.4 ± 0.2	7.7 ± 0.2	7.0 ± 0.3
	30% WHC	10.8 ± 0.1	7.1 ± 0.2	7.5 ± 0.2	6.4 ± 0.3
	60% WHC	9.7 ± 0.05	5.3 ± 0.3	6.7 ± 0.04	5.5 ± 0.2
	100% WHC	7.4 ± 0.3	3.5 ± 0.5	5.8 ± 0.2	5.0 ± 0.2

SL10: Soil with a depth of 0–10 cm; SL20: Soil with a depth of 10–20 cm; WHC: Water-holding capacity; sdw: soil dry weight. The values after the “±” represent the standard errors of 3 replicates.

## Data Availability

All data related to this research are included in this article. Any further information is available upon reasonable request.
